# Galvanoplastic Tooth Crowns

**Published:** 1888-06

**Authors:** C. E. Diehl

**Affiliations:** Pittsburgh.


ARTICLE VIII.

### GALVANOPLASTIC TOOTH CROWNS.

BY DR. C. E. DIEHL, PITTSBURGH.

The galvanoplastic art introduces into the dental laboratory an entirely new method for the manufacturer of gold tooth crowns, which shall be in every detail fac-smiles of the natural organs. A gold tooth crown, as made by the swagging process, is nothing more than a metallic ferrule, with one end closed resembling a lead-pencil top. Cusps struck up in dies and soldered to the ferrule do not give it a tooth-like appearance.

There seems to prevail, unfortunately, a wrong impression as to which are the most important features of a tooth.

The greatest efforts have been directed heretofore toward making the cutting or masticating surface resemble the natural tooth, at the expense of the more prominent features.

The cutting and masticating surfaces contribute little, if any toward making a tooth look real. We notice, in the the teeth of elderly persons, the cusps and cutting edges are much—in many cases altogether—worn away; yet the teeth present a perfectly natural appearance.

We have paid too much attention to the soles of the feet, neglecting the more important features—the face of the tooth, the neck, the curves from neck to cusps, and lateral lines.

To imitate these lateral lines by the swagging process seems practically impossible. Because, the circumference of the tooth is smallest at its neck; consequently, if a swagging tool were inserted into the crown and the gold made to conform thereto, the tool could not be withdrawn.

To overcome such a difficulty and many others, we have recourse to the Hydroplastic Art: The art of depositing metal through the agency of dynamic electricity and suitable metal solutions.

In the manufacture of a gold tooth crown by the galvanoplastic method, care and some experience are necessary. Each operator must learn what his battery and solutions are doing; whethere there is too little gold or too little cyanide of potassium in the bath and whether the current of electricity is too strong or not strong enough.

By carefully observing the actions of the bath, the operator will soon learn to interpret the signs which they indicate.

The first step as in other methods, is to get a perfect measurement of the root to be crowned. Next, to select a well-shaped natural tooth of porcelain crown, that will nearly fill the space which the finished gold crown is to occupy.

A mold is made of the natural tooth or porcelain crown in plaster of Paris; a two-piece mold will answer in nearly all cases. A casting gate is then cut, the mould fitted carefully together and a tooth pattern cast of a suitable fusible metal.

The tooth pattern is then fitted into the space furnished by the bite for length, and to the measurement of the root. This is easily done.

The fusible metal tooth pattern is quite soft; an ordinary rubber scraper is the only tool necessary. Finish with a series of graded grit powders.

Bore a small hole into the top of the tooth pattern; do not let the drill pierce the cutting or masticating surface.

The hole is for the insertion of a brass or copper wire two or three inches long. The wire will answer for a handle. We are now prepared to put the tooth pattern into the various baths, providing the pattern is perfectly clean and free from any oil or grease. Too much care cannot be paid to cleanliness in all galvanoplastic operations.

The first bath is one of copper, made of a simple salt of copper, the sulphate for example, or a double salt of copper—the cyanide of potsasium and copper. The formula will be given further on.

Fill a glass jar, holding about one pint, with the copper solution. Lay two copper rods across the top of the jar; attach to one by a copper wire, the positive pole of a galvanic battery, and to the other rod the wire proceeding from the negative pole—generally zinc. The little rods thus become, one positive the other negative.

The tooth pattern which is to receive the galvanoplastic deposit is suspended in the copper bath by the wire handle previously inserted from the negative rod, and is known as the cathode.

From the positive rod is suspended a plate of metal, such as the bath contains in solution, which at present is copper. This plate is known as the anode. It will be noticed that in a few moments the whole surface of the tooth pattern (cathode) will be coated with a fine film of copper, the thickness of which is entirely under the control of the operator. It is best, however, to allow the deposit to accumulate to the thickness of ordinary writing paper.

The copper-covered tooth pattern is now taken out of the copper bath, rinsed in clear warm water and transferred to a jar containing about one pint of gold solution. The little rods perform the same office as before, except the copper plate anode is dispensed with, and one of gold or platina substituted.

If the battery and solution is in harmony, metallic gold will be deposited on the copper-faced tooth pattern in a few moments. The process is continued till the required thickness of gold is attained.

It usually takes about twelve hours treatment to complete the deposition of gold. The time, however, is of little consequence, as the process goes on without much attention.

When the deposit is of sufficient thickness, the work is taken from the bath, washed, buffed and burnished. The wire handle is now withdrawn, the crown carefully picked up with tweezers and passed rapidly to and fro in a vessel of boiling water. The fusible metal will melt and run out, leaving within the grasp of the tweezers a beautiful gold crown. If the crown is a little large at the neck, it should be replaced in the gold bath, with the outside of the crown protected from a further deposit by "stopping off varnish;" gold will then deposit on the inside, thereby reducing the diameter at its neck

A galvanic battery may be made of almost any kind of elements, Smee, Danniel, Bunsen, Grove, Grenet, or Le Clanche.

### COPPER SOLUTIONS.

No. 1.—Stir a quantity of carbonate of copper in water, add cyanide of potassium, till the liquid becomes colorless.

No. 2.—Water 12½ parts, and cyanide of potassium, carbonate of soda, acetate of copper and bisulphite of soda, each ¼ part. Mix the acetate of copper in one-fifth of the water, add the water drop by drop at first. This salt is difficult to mix in the presence of a quantity of water. When the acetate of copper is dissolved, add the carbonate of soda. Add one-fifth more water and the bisulphite of soda. The liquid now becomes a dirty yellow. Add the remaining three-fifths of water and the cyanide of potassium. The result should be a colorless liquid. If after the cyanide is all dissolved and the liquid is not entirely colorless, the cyanide was not pure. To remedy the evil, add a little more cyanide. If we desire a perfectly limpid bath the solution must be filtered.

No. 3.—Sulphate of copper, 1 pound; and water 20 oz.

This bath must always be kept saturated, new supplies of the copper salt must be added. This is best accomplished by suspending a muslin bag containing the salt, in the solution. The addition of sulphuric, acetic or citric acid increases the conductivity and specific gravity of the solution, at the same time improving it materially.

### GOLD SOLUTIONS.

No. 1.—Chloride of gold, 1 part; and cyanide of potassium, 3 parts, distilled.

| | |
|---|---|
| No. 2—Phosphate of sodium, - - - | 6 parts. |
| Bisulphate of sodium, - - - - | 1 part. |
| Cyanide of potassium and | |
| Metallic gold, each - - - - | 1-10 part. |
| Distilled or rain water - - - - | 100 parts. |

The metallic gold is first reduced to a chloride

STOPPING OFF VARNISH.

| | |
|---|---|
| Yellow wax - - - - | 7 parts. |
| Resin (fine yellow) - - | 10 parts. |
| Sealing wax - - - - | 4 parts. |
| Peroxide of iron - - - | 2 parts. |

Chloro Gutta Percha.—Varnish of gutta-percha and chloroform will also do for "stopping off."

The surface of the anode should always correspond in size with the cathode.—*The Dental Office and Laboratory.*

---

ARTICLE IX.

